# Practical Observations on the Use of Sulphate of Quinine

**Published:** 1840-07

**Authors:** John W. Monette

**Affiliations:** Washington, Mississippi


### Art. II.—*Practical Observations on the use of Sulphate of Quinine.* By John W. Monette, M.D., of Washington, Mississippi.

In the 13th volume of the "American Journal of Medical Sciences," I gave my views of the medical properties of the sulphate of quinine, as a remedy in febrile affections, and of the proper cases for its use in the treatment of fevers of the remittent and intermittent type. In the article referred to, it is contended that the sulphate of quinine is a powerful *febrifuge* in fevers with increased action and tone, and acts somewhat analogously to antimony and other contra-stimulants; that it is by no means a direct stimulant to the circulation or nervous and cerebral energy; but is calculated to reduce the tone of both: that it is not by any means a tonic in the general acceptation of the term: that it is not adapted to the treatment of fevers attended with direct debility: nor to cases of debility without fever; and that it will produce pernicious effects in such cases if administered freely.

I propose in the present article to offer a few additional remarks in illustration of my views of the therapeutic properties of quinine. In this I shall cite a few cases and base my remarks upon them with the sole design of illustrating the views which I entertain. I am convinced, that much temporary inconvenience if not injury is often caused to patients, laboring under autumnal fevers and other febrile affections, by an injudicious administration of quinine; and that this injudicious administration proceeds from a mistaken idea of its precise medical properties: especially from the belief that it is purely tonic or stimulant. Neither of these properties pertains to the sulphate of quinine, unless it be indirectly, by reducing the febrile action and diathesis. In febrile affections attended with an irritable condition of the nervous system, and exhausted vigour of animal action, quinine will be decidedly prejudicial. In these cases it increases the nervous irritability, impairs cerebral energy, and produces great precordial distress. I know several persons of nervous temperament who suffer so much from a single dose of quinine, that no persuasion would induce them to repeat it: one gentleman in particular is thrown into a perfect delirium by a full dose of quinine, even in the state of fever, and much more so in the absence of fever. Two cases came under my observation last summer in which death was caused in a few hours by only two doses of quinine. The patients were two black children, one five, and the other six months old. They were taken with a catarrhal bilious fever about the 10th of September, when it prevailed epidemically in this vicinity among the infants and children. These two infants had been sick four or five days, had taken eight or ten small doses of calomel, which had excited and kept up copious yellow and green mucous discharges from the bowels; and the general nervous and cerebral irritation was augmented, and the febrile action reduced to the irritable kind. At this stage I saw them; prescribed mild demulcents, gentle anodynes, fomentations to the abdomen, and gentle saline laxatives after the irritable condition of the bowels was allayed. On the third day after I saw them, they were clear of fever and apparently in a fine condition for convalescence. In this condition, with a view of preventing fever, each of them took one grain of quinine and half a grain of Dover's powder at 2 o'clock, and repeated it at 5 o'clock. Soon after the first dose, the fever appeared to rise, the pulse became active and throbbing; the head hot, and pulsating, with general uneasiness. All these symptoms increased soon after the second dose; and became greatly aggravated about 7 o'clock, attended with partial spasms. One died at 9 o'clock, and the other in half an hour afterwards.

*Remarks.*—At the time the first dose of quinine was given, these children were in a fine state for convalescence without any medicine, and would in all probability have rapidly recovered under the occasional exhibition of a very small portion of weak brandy toddy, with a few drops of paregoric, or essence of peppermint in it; and with an occasional mild laxative. The quinine in very small quantities, one-eighth to one-fourth of a grain, might have been beneficial: but the condition of the little patients was not such as to bear a full dose. Adults in our country are sometimes, during convalescence from bilious fevers, in precisely analogous circumstances, especially females; and in them quinine administered in the same proportion would be almost as detrimental. But in them a consciousness of its pernicious effects would cause them to refuse its further administration. In such cases, if the tone of the system were not impaired, and if the nervous temperament did not predominate, the use of quinine combined with some aromatic would be applicable: but otherwise the infusion of cinchona and ginger, or weak toddy is decidedly preferable.

The above is evidently the experience of M. Vulpes, of Naples, when he describes the sulphate of quinine as "more irritating than the cinchona." Yet Dr. Eberle, (Practice, vol. 1, p. 73,) in commenting upon the experience of M. Vulpes, says, he has never perceived such difference in the action of the two articles. Yet Dr. Eberle (Ibid, p. 76,) admits that he has seen pernicious effects from large doses in the cold stage of intermittents; and, that he suspects that certain cerebral irritations in a lady of delicate constitution, and in a lad, were attributable to its improper use. These effects I am confident will not be produced in the robust and the athletic, whether they have fever or not. Dr. Eberle however thinks quinine a good substitute for barks, and much more convenient when bark is rejected by the stomach. On this point I remark, that quinine will disagree with the nervous system as often as the bark does with the stomach; but the cases in which quinine generally disagrees, are those of persons of nervous temperament, and irritable condition of the nervous system.

Very little testimony of any kind in relation to quinine is to be obtained from Dr. Eberle's Practice of Medicine; and it is somewhat surprising that he does not recommend quinine as a remedy in fevers, either continued, remittent, typhus, or bilious. He recommends it principally in intermittents, and even in those cases, he appears to be without any definite views as to its specific action, other than as a tonic or substitute for bark. Throughout the whole work he confounds the two articles, cinchona and sulphate of quinine, as possessing the same properties, and applicable in the same cases. There is no man whose authority in medicine I respect more than that of Dr. Eberle, in matters pertaining to therapeutic practice: but in regard to the use of quinine I am satisfied he has not made such use of it as would enable him to speak of it in clear and definite terms.

To illustrate my views of the pernicious effects of quinine in persons of delicate constitution, nervous temperament, and debilitated condition, I will give the following case, which came under my observation in the spring of 1837.

Case.—Mrs. L. F. æt. 28 or 30 years, of temperament lymphatic and nervous, strongly marked. During the latter months of gestation with her third child, she became greatly afflicted with deranged functions in all the organs of assimilation; such as were indicated by pyrosis, cardialgia, anorexia, and even bulimia, alternately, and an inability to retain anything on her stomach. These symptoms became so aggravated towards the termination of gestation, that for six weeks previous to her accouchement, she was confined to her bed and room. Various antacids, carminatives and such like remedies were used without relief; venesection was used freely several times, and, to say the least of it, without benefit. She became weak, pale, and emaciated, although with a kind of œdematous fulness of features. In this state the pulse became weak, quick, and irritable; the brain became irritable and very sensible to the slightest degree of light and sound. In this state she was delivered of a fine healthy son. She passed through the stage of labor without any untoward circumstance, but her condition became daily more precarious and distressing. I first saw her on the eighth day after her accouchement, in the following condition: viz.—The lochiæ were profuse and watery; there was a profuse secretion and frequent discharge of thin yellow bile; the pulse weak, quick and irritable; the stomach frequently rejected all ingesta; the senses of sight and hearing were so acutely sensitive that the room was kept perfectly dark, and the lightest walking in the stocking feet over a carpeted floor gave her great pain, as did also the most moderate whispers in the opposite side of a large room. During the last eight days she had been under the constant attendance of two physicians, and had used freely of magnesia, blue mass, calomel to free ptylism, besides bloodletting twice before the mercurials. Sulphate of quinine was also alternated, with occasionally a small portion of morphia. Nevertheless, all the distressing symptoms had become daily more alarming; and when I first saw her she was unable to raise her head from the pillow; the tongue and face as pale as marble. The last medicines which she had taken were small doses of quinine and blue pill, to the exclusion of the small portions of morphia which she had been taking previously.

In this state I advised the discontinuance of the blue pill and quinine, and to substitute aromatic stimulants and morphia in small quantities; but to satisfy the physician in previous attendance I consented to continue the quinine in doses of 1 gr. every six hours, besides other remedies. Having remained three days and nights without intermission, I had the best opportunities for observing the effects of every article given. About one hour after each portion of the quinine was taken, although in so small a quantity, the nervous irritation became aggravated. The portion of quinine was occasionally omitted to see whether the accompanying change would take place; but the aggravated state of nervous sensibility and irritation did not occur without the quinine, and it was finally dispensed with entirely. Having, in cases somewhat similar, obtained good effects from the oxide of bismuth, I determined to give it a trial in this case. I accordingly gave a powder composed of 3 grs. ox. bism., ¼ gr. ipecac and 1-6 gr. morphia every three hours until the cerberal and general irritation was in a measure composed, and afterwards *pro. re nâta.* The result for several days was such as gave the most flattering prospect; although about ten days afterwards a change occurred in her disease, and she died in my absence.

*Remarks*—I have given this as a minute detail of a most aggravated case of that kind of disordered action, in which quinine is decidedly pernicious. From my observations in this and other cases, I am convinced that had this patient taken 3 grs. of quinine every three hours, instead of 1 gr. every six hours, it would have terminated her existence in a few hours.

Dr. Cartwright seems to have used quinine as a direct febrifuge in 1823–'4–'5, although that was the experimental period of quinine, when it was generally considered only as a tonic. In his essays, published in the Medical Recorder,* he appears to have used it as a febrifuge, to sustain febrile action, and to develope suppressed excitement, especially in pneumonia biliosa. However, in some cases it appears to have had a pernicious effect throughout its administration. Mr. Dearborne's case is one of these. See Medical Recorder, vol. x. pp. 48 and 49, &c.

The direct effects of quinine in improper cases, is to produce a collapse of cerebral and nervous energy—a want of nervous concentration, if the expression may be allowed, a diminution of voluntary action, both mental and corporeal; a confused state of intellect, attended with *tinnitus aurium*, giddiness, vertigo, pain in the head; a feeble, quick or irritable pulse, cerebral irritation, and nervous tremors in the mus-

---

* Volume X.

cular system. Whether these effects be produced by cerebral congestion, or by a direct diminution of cerebral energy, is of no importance to enquire. The fact is all that is necessary to be known in regulating its administration.

*Congestive fevers* appear to result from a collapse of nervous energy, an overwhelming of the whole recuperative powers of the system, whence all efforts to establish excitement and reaction by the powers of animal life unassisted, fail, and the fatal collapse ensues. This powerful impression results from the greater force of the morbific agent, or from the coincidence of a greater number of auxiliary circumstances attending the condition of the individual attacked, or from the greater degree of susceptibility of the system to the morbific influences, and the less vigorous recuperative powers of constitution in the individual attacked. In strong athletic constitutions, with strong powers of reaction, the excitement becomes open and general; and when no organ is implicated by previous disease there will be very little if any local determinations. In these constitutions if there be partial oppression of circulation, quinine alone or combined may be useful: but in the former it is injurious. In the former, the tone, or the recuperative powers of the system are only oppressed, not direcetly reduced; hence it is in such cases generally, that we find quinine has been used with benefit in the early stages of fevers, before local determinations and engorgements were established, or the functional action of any organ had become permanently deranged. In these cases, we conceive the poisonous ferment in the system to be neutralized to a certain extent, before the establishment of those functional and structural derangements, which, until removed, in a great degree, preclude the use of quinine.

Thus in cases of great gastro-intestinal irritation, indicated by a clean, dry, smooth tongue; or a tongue dry, red, and chapped, or dry, brown, and rough, the quinine is useless if not injurious; because the existing intestinal irritation is sufficient to keep up a low grade of fever, even were the original exciting causes withdrawn: and the irritated mucous surfaces, in contact with the quinine, would be further irritated by that medicine, whereby the general sum of the morbid symptoms would be aggravated. This grade of gastro-intestinal irritation scarcely ever presents in the first stages of any fever, but supervenes in continued febrile action, where the mucous membrane receives additional irritation from the repeated irritating effects of purgatives, or other acrid remedies. In these cases quinine unquestionably accelerates that stage called low typhoid fever, accompanied by cerebral irritation and incoherent delirium.

The proximate cause of fever appears to be some essential morbific action or ferment in the circulation, and is modified in its action and operation upon the system by the circumstances of climate, locality, temperament, habits, and other extrinsic circumstances. Other contingent circumstances often cause the particular location of the disease upon particular organs or tissues. The peculiar grade of action in each of these organs and tissues is often modified, in like manner, by circumstances which elude our observation. In a sound constitution, where no organ is more disposed to yield than others, the action is confined to the general circulation and secretory organs, and developes itself in what is termed idiopathic fever. Yet in this grade of fever, a depravation of fluids and secretions often supervenes; and these secretions again operate as new exciting causes of fever, or new sources of morbid irritation.

Although *quinine* is a strong febrifuge—an antidote to this morbific *virus*, yet its action is confined to the subduing of that morbid action, while there is no local source of irritation. It will not remove morbid and acrid secretions that proceed from morbid action, and serve as a new source of irritation. It will not remove inflammation, which may have resulted from general excitement and the predisposition of a weak organ, in any of the great cavities. It will not remove morbid sensibility and irritability which has supervened in any organ or tissue, from the irritation of morbid secretions, or from local determinations. It will not reduce general febrile action proceeding from the primary morbific agent or miasm, while at the same time all these new sources of febrile irritation are in full operation. Hence, in using sulphate of quinine in fevers, it becomes necessary to reduce, in some degree, the violence of arterial action by the different modes of depletion and narcotics; to remove the irritating secretions from the alimentary canal, and allay with narcotics the irritation already excited. It is necessary to relieve the determination to any particular organ, by antiphlogistic remedies and counter-irritants. It is necessary to develope the circulation and produce open excitement, where it is oppressed and attended with local congestions. It is necessary to allay by opiates and anodynes, functional excitement or irritation, which may be superinduced in the train of febrile symptoms; such as profuse secretion of bile, or profuse serous secretion from the mucous surfaces of the intestinal canal. In short it is necessary to place the system in a state of simple idiopathic fever, as nearly as possible, before the free administration of *quinine* will be beneficial. Upon this principle we see why this medicine may be given without producing any beneficial results, even where it does not produce positive injury.

Upon this principle we insist that the sulphate of quinine is not a tonic strictly speaking; although it may apparently fulfil the office of a tonic in certain cases, where a slow febrile action is the principal cause and source of the debility. In such cases quinine will appear to act as a tonic; while its real action is to reduce the febrile action concealed in the system, and permit the recuperative powers of the system to restore the natural action in all the functions deranged.

Dr. Perrine, from Campeachy, in a letter to Dr. Drake, published in the Western Medical Journal, appears to entertain the same views of the febrifuge properties of quinine, that we do. In relation to the opposition shown to large doses of this medicine, he says, neither will they "believe that ten grains of quinine will reduce an elevated pulse, and moisten a dry skin, while one grain-doses will irritate both: neither can they believe that quinine can be used like calomel in fevers, in connexion with bleeding and other antiphlogistic remedies." (See number for January, 1834, p. 330.) As to his views in relation to the "irritation of large doses," as well as his views in regard to the use of quinine in the malignant cholera, I am compelled to differ in some respects. Yet he appears to have ascertained, that the properties of sulphate of quinine are essentially febrifuge—and adapted to the treatment of fevers of open excitement.

*December*, 1839.

